# Hydroxychloroquine improves insulin sensitivity in obese non-diabetic individuals

**DOI:** 10.1186/ar3868

**Published:** 2012-06-07

**Authors:** Emileigh Mercer, Laura Rekedal, Rajesh Garg, Bing Lu, Elena M Massarotti, Daniel H Solomon

**Affiliations:** 1 Division of Rheumatology, Brigham and Women's Hospital/Harvard Medical School, 75 Francis Street, Boston, MA 02115, USA; 2 Division of Endocrinology, Brigham and Women's Hospital/Harvard Medical School, 75 Francis Street, Boston, MA 02115, USA

## Abstract

**Introduction:**

Hydroxychloroquine (HCQ) is a common disease modifying therapy for the treatment of rheumatoid arthritis (RA). Prior research suggests that HCQ may reduce the risk of diabetes mellitus in patients with RA. To investigate the mechanism of this effect, we examined the effect of HCQ on insulin resistance, insulin sensitivity, and pancreatic β-cell secretion of insulin in non-diabetic, obese subjects.

**Methods:**

We recruited 13 obese, non-diabetic subjects without systemic inflammatory conditions for an open-label longitudinal study of HCQ 6.5 mg per kilogram per day for six weeks. Subjects underwent an oral glucose tolerance test at three time points: 0 weeks (pre-treatment with HCQ), 6 weeks (at the end of the HCQ treatment), and 12 weeks (6 weeks post HCQ-treatment). The Matsuda Insulin Sensitivity Index (ISI), HOMA-IR, and HOMA-B were compared across time-points.

**Results:**

The mean age of the cohort was 49 years, 77% females and median body mass index was 36.1 kg/m^2^. After 6 weeks of HCQ therapy, ISI increased from a median (interquartile range) of 4.5 (2.3-7.8) to 8.9 (3.7-11.4) with a p-value of 0.040, and HOMA-IR decreased from a median of 2.1 (1.6-5.4) to 1.8 (1.02-2.1) with a p-value of 0.09. All these variables returned toward baseline at week 12.

**Conclusion:**

HCQ use for 6 weeks in non diabetic obese subjects was associated with a significant increase in ISI and trends toward reduced insulin resistance and insulin secretion. These data suggest that HCQ, a common medication used to treat RA, possesses beneficial effects upon insulin sensitization. Further study of the insulin sensitizing effects of HCQ in patients with RA is warranted.

## Introduction

Individuals with several different rheumatic diseases carry a greater risk of developing cardiovascular disease (CVD)[1-4] . It is likely that the systemic inflammation underpinning these conditions contributes to an increased risk of CVD [5]. One possible link between the systemic inflammation of rheumatic diseases and an increased risk of CVD is worsening of insulin resistance. Insulin resistance is a common risk factor for both CVD and diabetes mellitus (DM) [6]. Insulin resistance refers to impaired insulin sensitivity and glucose metabolism, and commonly precedes development of DM [6]. Additionally, insulin resistance represents one facet of the metabolic syndrome, a constellation of risk factors that predict CVD events [1,2,7,8].

Insulin resistance occurs more frequently in systemic rheumatic diseases, such as rheumatoid arthritis (RA) and systemic lupus erythematosus (SLE) [9,10]. In rheumatic disease patients, systemic inflammation appears to act directly on insulin and glucose metabolism through elevated levels of TNFα and IL-6 [9,11-13]. Several pharmacologic treatments for rheumatic diseases have been associated with improvements in insulin and glucose metabolism. Anakinra, an IL-1 antagonist, effectively reduced glycated hemoglobin in patients with type 2 DM, and TNFα antagonists have improved insulin resistance in patients with RA or ankylosing spondylitis [11-14].

Hydroxychloroquine, a US Food and Drug Agency-approved medication for SLE and RA, has also been shown to improve glycated hemoglobin in patients with poorly controlled type 2 DM [15,16]. A cross-sectional study of CVD risk factors among women with SLE or RA reported better glycemic control, in multiple measures, during HCQ use [17]. In two large epidemiological studies of patients with RA, an association was noted between HCQ use and a reduced risk of developing DM [18,19]. Furthermore, animal models suggest that HCQ may retard insulin degradation [20,21]. However, we find no human studies in the published literature that examine the effect of HCQ on insulin and glucose metabolism.

In light of this background, we pursued a pilot study among non-diabetic obese subjects without a known systemic inflammatory condition. We assessed HCQ's effect on insulin sensitivity and secretion during a short-term study and hypothesized that insulin sensitivity would improve during HCQ administration.

## Materials and methods

### Study population

Thirteen adult subjects were recruited from the community. All aspects of this study were approved by the Partners Institutional Review Board (IRB) at Brigham and Women's Hospital. Additionally, each patient signed an informed consent form that was obtained according to the Declaration of Helsinki and approved by the IRB at Brigham and Women's Hospital. All had a body mass index (BMI) ≥ 30 kg/m^2 ^and no history of DM. Subjects were excluded if there was current oral corticosteroid use, or a history of neuromuscular disease, psoriasis, chronic inflammatory intestinal disorders or eye disease (with the exception of cataracts or glaucoma). Additionally, confirmation of normal liver and kidney function testing was required before drug administration. The Partners HealthCare System IRB approved all aspects of this study.

### Intervention

Subjects were administered a six-week course of HCQ (6.5 mg/kg) over the course of the study. At the baseline visit, subjects were given a three-week supply of HCQ and complete instructions on daily administration. During the safety visit (week 3 following baseline), subjects were given their second three-week supply of HCQ. Subjects were then instructed to stop taking HCQ at week 6, and return any unused study drug to the study staff. A record was kept of each subject's pill counts and reminder phone calls were made to keep subjects as compliant as possible. All subjects were closely monitored and in contact with study staff throughout the twelve-week protocol.

### Study procedures and data collection

After eligibility was confirmed through screening procedures, all subjects completed a total of four study visits and two phone conversations with study staff over the course of twelve weeks (see Additional file 1 for schedule of visits). At the baseline visit, the first of three oral glucose tolerance tests (OGTT) was performed with blood samples collected every 30 minutes for 120 minutes to measure the primary outcome of interest, insulin sensitivity index (ISI). The ISI was calculated based on the equation of Matsuda [22] and the area under the curve of insulin and homeostasis model assessment-estimated insulin resistance (HOMA-IR) were also calculated [23]. The formulas for each of these calculations are as follows.

Matsuda insulin sensitivity index:

$$\text{IS}{\text{I}}_{\text{(Matsuda)}}=\frac{10000}{\sqrt{{\text{G}}_{\text{0}}\times {\text{I}}_{\text{0}}\times {\text{G}}_{\text{mean}}\times {\text{I}}_{\text{mean}}}}$$

ISI, insulin sensitivity index; G_0_, fasting plasma glucose (mg/dL); I_0_, fasting plasma insulin (mIU/L); G_mean_, mean plasma glucose during OGTT (mg/dL); I_mean_, mean plasma insulin during OGTT (mIU/L)

HOMA-IR:

$$\text{HOMA - IR = }\frac{\text{Glucose}\times \text{Insulin}}{405}$$

HOMA-B:

$$\text{HOMA}-\beta =\frac{360\times \text{Insulin}}{\text{Glucose - 63}}\%$$

Additionally, secondary laboratory outcomes were measured, including enzymatic tests for total cholesterol, high density lipoprotein (HDL), calculated low density lipoprotein (CLDL), and triglycerides. Analysis of C-reactive protein (CRP) was completed by latex immunoturbidimetry, of C-Peptide by radioimmunoassay (Siemens, Los Angeles, CA, USA) and of IL-6 by immunoassay (Access Chemluminescent Immunoassay by Beckman Coulter, Fullerton, CA).

In addition, BMI, blood pressure, and muscle strength were recorded at the baseline, six- and twelve-week visits. One week after the baseline visit, subjects were contacted by the study team to screen for potential adverse effects of the HCQ. If any potential HCQ-related events had occurred, the principal investigator was immediately notified and the subject was contacted. Three weeks following baseline, subjects had an in-person safety interview with a member of the study team to further screen for potential adverse effects of HCQ; BMI, blood pressure, and muscle strength were all once again recorded. At six weeks after baseline, subjects returned for their second OGTT, as well as measurement of secondary outcomes, BMI, blood pressure, and muscle strength screening. At nine weeks after baseline, a second phone conversation was conducted to screen for any other effects since stopping HCQ. Finally, twelve weeks after baseline subjects underwent their final OGTT, as well as secondary laboratory outcome measures, BMI, blood pressure, and muscle strength screening.

### Statistical analyses

Descriptive statistics such as minimum, maximum, range, median and interquartile range **(**IQR) were used to describe the primary outcome (ISI) and secondary outcomes. The primary outcome, change in ISI levels, was calculated by comparing pre-treatment (baseline) with and during treatment (week 6), and between week 0 and week 12 using Wilcoxon signed-rank tests. Similar analyses were conducted for HOMA-IR and the secondary laboratory outcomes. All *P*-values were calculated with a two-sided significance level of 0.05. Data analyses were performed using SAS 9.1.2 (SAS Institute, Inc, Cary, North Carolina).

## Results

Of the 111 potential subjects who underwent pre-screening, 37 were interested and pursued formal screening, and 13 of the 37 subjects met the inclusion criteria and followed through with all study visits. Subjects who were excluded after formal screening included 17 who decided not to participate, 3 who were not eligible, 3 who did not meet the laboratory or medical screening criteria, and 1 who did not pass the baseline eye screening.

Among the 13 enrolled subjects, the median age was 49 years with participants ranging in age from 24 to 71. The 13 participants included 10 women. The median BMI of the subjects was 36.1 kg/m^2^. Baseline characteristics were within the normal range for laboratory parameters (Table 1).

**Table 1:** Baseline characteristics of thirteen study subjects

	Mean (± standard deviation) or median (interquartile range)
Age, years	49 (± 15)
Female gender	77%
Body mass index, kg/m^2^	36.1 (30.7 - 38.4)
Blood pressure, diastolic	121.5 (± 5.2)
Blood pressure, systolic	74.7 (± 7.1)
Serum glucose, mg/dl	92 (77 - 95)
Serum insulin, μIU/mL	10.44 (6.89-22.7)
Total cholesterol, mg/dL	165 (146 - 181)
Low density lipoprotein cholesterol, mg/dL	91 (79 -120)
High density lipoprotein, mg/dL	45 (39 - 57)
Triglycerides, mg/dL	85 (64 - 98)
C-reactive protein, mg/L	2.80 (1.3-4.6)

**Figure 1:**
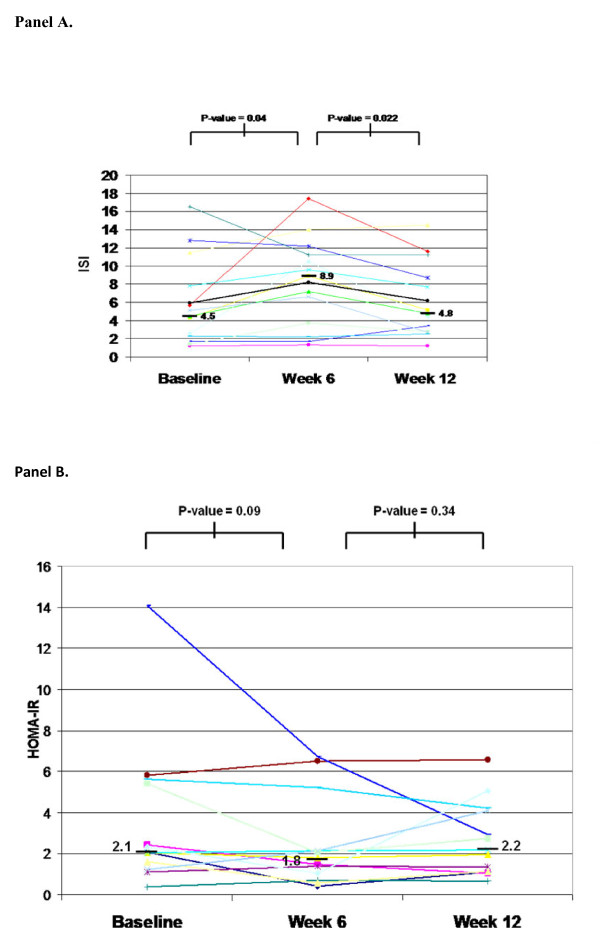
**Insulin sensitivity and insulin resistance**. This figure depicts values for (**A**) the Insulin Sensitivity Index (ISI), and (**B**) the homeostasis model assessment-estimated insulin resistance (HOMA-IR) for each of the 13 study subjects at baseline, 6 weeks, and 12 weeks. Medians are also indicated as the bars with adjacent values.

**Figure 2:**
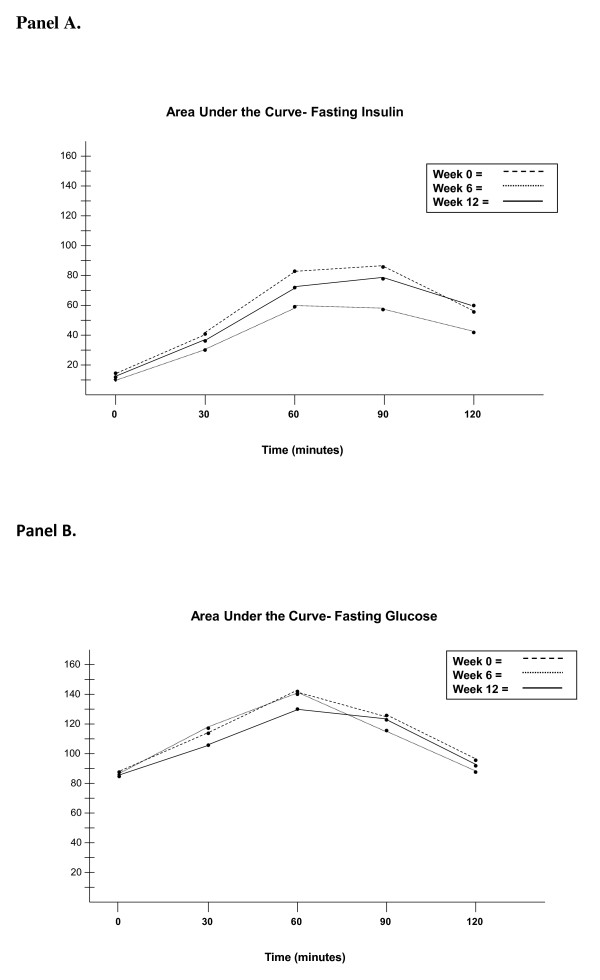
**Area under the curve, glucose**. This figure illustrates the area under the curve (AUC) for (**A**) glucose and (**B**) insulin, during the oral glucose tolerance tests at weeks 0, 6, and 12. The AUC for glucose was 240 ± 88 (week 0), 232 ± 92 (week 6) and 227 ± 71 (week 12). The differences in AUC for glucose were not statistically significant (week 0 to week 6, *P *= 0.45, and week 6 to week 12, *P *= 0.89). The AUC for insulin was 134 ±125 (week 0), 95 ± 91 (week 6) and 123 ± 91 (week 12). The differences in AUC for insulin between week 0 to week 6 (*P *= 0.09) and week 6 to week 12 (*P *= 0.021) suggest an important trend.

The median (IQR) increase in ISI during HCQ treatment between baseline and week 6 was from 4.5 (2.3 to 7.8) to 8.9 (3.7 to 11.4) with a *P*-value of 0.040 (Figure 1a). We observed a return toward baseline in ISI by week 12 (*P *= 0.45 comparing baseline to week 12). HOMA-IR was also examined, and decreased during HCQ treatment between baseline and week 6 from a median of 2.1 (1.6 to 5.4) to 1.8 (1.02 to 2.1) with a *P*-value of 0.09 (Figure 1b), but this decrease was not statistically significant. Between week 0 and week 12 (post-HCQ), HOMA-IR trended back towards baseline (*P *= 0.64 comparing baseline and week 12). The areas under the curve for glucose and insulin were plotted (Figure 2) and showed stability for glucose, but reductions in insulin at week 6 during HCQ.

For measurement of the secondary outcomes, we found small differences between weeks 6 and 12 for total cholesterol, CLDL and HDL. However, no differences were observed in CRP, C-peptide, or IL-6 (Table 2).

**Table 2:** Secondary outcomes (fasting) from study

	Median (interquartile range)			*P*-values	
	0 weeks	6 weeks	12 weeks	0-6 weeks	6-12 weeks
Total cholesterol, mg/dL	165 (146, 181)	166 (130, 178)	173 (146, 184)	0.18	0.0002
High density lipoprotein, mg/dL	45 (39, 57)	48 (3, -57)	47 (43, 60)	0.95	0.02
Low density lipoprotein, mg/dL	91 (79, 120)	83 (70, 102)	89 (81, 126)	0.15	0.003
Triglycerides, mg/dL	85 (64, 98)	75 (52, 116)	81(71, 98)	0.33	0.95
C-reactive protein, mg/L	2.80 (1.3, 4.6)	2.98 (1.7, 3.4)	1.91(1.1, 6.3)	0.47	0.96
Fasting insulin, μIU/mL	10.44 (6.89, 22.7)	9.37 (4.27, 10.26)	10.48 (6.87, 17.01)	0.09	0.45
Glucose, mg/dL	92 (77, 95)	88 (76, 97)	86 (80, 97)	0.64	0.72
C-peptide, ng/mL	3.81 (2.21, 4.35)	3.05 (2.32, 4.20)	3.30 (2.15, 4.37)	0.49	0.35
Interleukin-6, pg/mL	2.90 (2.15, 3.92)	3.06 (2.13, 3.84)	3.12 (2.64, 4.03)	0.81	0.62

## Discussion

This pilot intervention study demonstrates that six weeks of HCQ treatment improves insulin sensitivity in obese non-diabetic subjects without a known systemic inflammatory condition. Animal models, randomized controlled clinical trials, and two epidemiologic studies have all shown that HCQ positively affects insulin and glucose metabolism [15-21]. We found a statistically significant increase in ISI after 6 weeks of HCQ and a decrease in ISI toward baseline after stopping HCQ. The area under the curve analysis agrees with the ISI analysis, in that less insulin maintained similar serum glucose levels. Reduction in HOMA-IR during HCQ also suggests improved insulin sensitivity, in that less insulin was required to control glucose at week 6. Statistical significance was not established for these results, but the small sample size could have produced a false negative error. There were no important changes in secondary outcomes, such as CRP or IL-6, during this short-term study, and BMI was consistent.

While this pilot study does not allow us to determine the clinical relevance of HCQ's effect on insulin sensitivity, this degree of improvement in insulin sensitivity may translate into a reduced risk of DM, as suggested by two large epidemiologic studies among persons with rheumatoid arthritis [18,19]. While we did not study subjects with rheumatic disease, we did look at obese individuals with increased baseline insulin resistance and an elevated baseline CRP, similar to persons with rheumatic disease. With HCQ's benefit as a disease-modifying antirheumatic drug, if it also improves insulin sensitivity in persons with rheumatic disease, it may be beneficial to maintain HCQ use in the rheumatic disease population.

The small sample size and short duration of drug administration limits this study, which should be viewed as hypothesis generating. Also, in Figures 1a and 1b, two different study subjects who were potential outliers were included in the statistical analysis, and the results remained statistically significant. It is important to take note of these skewed data points, even though it cannot be determined whether they are due to individualized responses to HCQ or the possibility that the subject may not have fasted before testing despite affirming to the study staff that they had.

The use of the Matsuda ISI as a surrogate measure for a clinical outcome, such as DM, is another limitation of this study. However, a paper on the current approaches to measuring insulin sensitivity suggests that the Matsuda ISI is highly correlated with results from the gold standard of metabolic testing, the euglycemic insulin clamp [24]. Additionally, the Matsuda ISI provides a dynamic measure for analyzing both glucose uptake and insulin secretion in response to a challenge, and has proved to be an accurate predictor of DM in epidemiologic studies [24]. Clinical studies among a variety of patient populations including those with DM, obesity, and stroke have successfully used ISI to evaluate a change in insulin sensitivity after undergoing drug treatment [25-29]. Studies using metformin, moxonidine, glyburide, glargine insulin, rosiglitazone and pioglitazone have reported changes in ISI ranging from 0.14 to 1.18 after treatment [25-29]. In this study, even larger changes in ISI after HCQ treatment were reported.

In this small pilot study we found that during HCQ use, obese non-diabetic subjects experienced a significant benefit in insulin sensitivity. No concurrent evidence of improvement in inflammatory markers was observed (for example, CRP and IL-6 did not change). This argues for a direct effect of HCQ on insulin metabolism-reduced degradation or enhanced activity at the receptor level, rather than an indirect effect through reduced inflammation. An important next step in this line of investigation would be a larger and longer study examining the effect of HCQ on insulin sensitivity, focusing on subjects with systemic inflammatory conditions, a population at an increased risk for insulin resistance and DM [30,31]. If HCQ improves insulin sensitivity in people with systematic rheumatic disease, a diabetes prevention trial should be considered for high risk patients with RA or SLE.

## Abbreviations

BMI: body mass index; CLDL: calculated low density lipoprotein; CRP: C-reactive protein; CVD: cardiovascular disease; DM: diabetes mellitus; HCQ: hydroxychloroquine; HDL: high density lipoprotein; HOMA-IR: homeostasis model assessment-estimated insulin resistance; IL-6: interleukin-6; IQR: interquartile range; IRB: internal review board; ISI: insulin sensitivity index; OGTT: oral glucose tolerance test; RA: rheumatoid arthritis; SLE: systematic lupus erythematosus; TNFα: tumor necrosis factor alpha.

## Competing interests

Dr. Solomon receives salary support from research contracts with Amgen, Abbott, and Lilly. He has also participated in an educational course sponsored by Bristol Myers Squibb. He has unpaid roles (DSMB and Executive Committee) on two Pfizer-sponsored trials. Dr. Massarotti is a consultant for Bristol-Myers Squibb, Pfizer, UCB, Roche, Medco Health Solutions, Human Genome Science, Policy Analysis Incorporated. She is an investigator for EMD Serono, BMS, and the NIH.

## Authors' contributions

EM and LR collected data, analyzed the data, and drafted the manuscript. RG conceived of the project and analyzed the data. BL analyzed the data and drafted the manuscript. EMM also conceived of the project and analyzed the data. DHS conceived of the project, collected data, analyzed the data, and drafted the manuscript. All authors revised and approved the final manuscript.

## Supplementary Material

Additional File 1 **Appendix Table 1 Schedule of study visits and phone calls**. A descriptive table of all study visits and data collected at each visit. Microsoft Word 3 column table. Click here for file
