# Supplementary material for: Hydroxychloroquine improves insulin sensitivity in obese non-diabetic individuals
Source: Arthritis Res Ther. 2012 Jun 7;14(3):R135. doi: 10.1186/ar3868 (PMC3446518; doi:10.1186/ar3868)
Supplement: Additional File 1 — Appendix Table 1 Schedule of study visits and phone calls. A descriptive table of all study visits and data collected at each visit. Microsoft Word 3 column table. [file ar3868-S1.DOCX]

Appendix Table 1. Schedule of study visits and phone calls

| Week | Visit Format | Study Procedures Performed |
| --- | --- | --- |
| -2/Screening Visit | In-person visit | Screening inclusion check  Confirmation of Eye Health  Screening labs* |
| 0/Baseline | In-person visit | -BMI, blood pressure, muscle strength  -Oral glucose tolerance test†  -Secondary laboratory outcomes‡  -Administer first three-week course of HCQ |
| 1 | Phone call | -Screen for adverse events with HCQ |
| 3 | In-person visit | -BMI, blood pressure, muscle strength  -Screen for adverse events with HCQ  -Administer second three-week course of HCQ |
| 6 | In-person visit | -BMI, blood pressure, muscle strength  -Oral glucose tolerance test†  -Secondary laboratory outcomes‡ |
| 9 | Phone call | -Screen for adverse events with HCQ |
| 12 | In-person visit | -BMI, blood pressure, muscle strength  -Oral glucose tolerance test†  -Secondary laboratory outcomes‡ |

* Screening labs include, Creatinine, ALT, AST, and a Complete Blood Count

†Oral glucose tolerance test, OGTT, completed by drawing 8.5ml blood every 30 min (5 draws total) after administration of glucose solution; done after ≥10 hours fasting.

‡Secondary outcome labs include Total Cholesterol (TCH), Calculated low density lipoprotein (CLDL), High density lipoprotein (HDL), Triglycerides (TRI), and C-reactive protein (CRP), C-peptide and IL-6.
